# Socio-psychological Study of COVID-19 Pandemic among Healthcare Workers in a Medical College of Nepal: A Descriptive Cross-sectional Study

**DOI:** 10.31729/jnma.5594

**Published:** 2021-02-28

**Authors:** Sabin Bahadur Zoowa, Lochana Shrestha, Leela Paudel, Ganesh Bhandari, Suhail Sapkota, Bibek Timilsina

**Affiliations:** 1 Department of Community Medicine, Nepalese Army Institute of Health Sciences, Sanobharyang, Kathmandu, Nepal; 2 HAMS hospital, Kathmandu, Nepal

**Keywords:** *anxiety*, *COVID-19*, *depression*, *health care workers*, *stress*

## Abstract

**Introduction:**

Health care workers experienced considerable psychological distress as a result of COVID-19 due to providing direct patient care, quarantine or self-isolation, and lockdown experience. They are front line workers handling the patients and are at greater risk than others. This study aims to determine the socio-psychological impact of COVID-19 pandemic among Nepalese Army Institute of Health Sciences health care workers.

**Methods:**

A descriptive cross-sectional study from different institutions of the Nepalese Army Institute of Health Sciences from May 2020 to July 2020 was conducted. Ethical approval was taken from the Institutional Review Committee, Nepalese Army Institute of Health Sciences. A total of 212 responses were collected through Google form along with the Depression, anxiety, stress and scale-21 to assess the level of Depression, Anxiety, and Stress. Analysis of the data was done using SPSS version 22.

**Results:**

Respondents with extremely severe depression, anxiety, and stress was found to be 16 (7.5%), 24 (11.3%), and 4 (1.95%) respectively. Most of the respondents do not have travel history, but 6 (2.8%) and 28 (13.2%) have direct and indirect contact respectively with the COVID patients. Daily activities such as food intake, the workload at home, and relationships with family members were increased. 208 (98%) have followed preventive measures such as mouth mask, hand washes, and physical distance.

**Conclusions:**

COVID-19 pandemic has imposed a significant level of depression, anxiety, and stress on health care workers caring for infected patients, with their main concern being the risk of transmitting the infection to their families or acquiring it themselves.

## INTRODUCTION

The coronavirus disease-2019 (COVID-19) is spreading rapidly worldwide which was considered a public health emergency by the World Health Organization and declared a pandemic by March 2020.^1^ Infections and deaths due to COVID-19 have been increasing exponentially, and its worldwide impact has been seen among health care workers (HCWs) in relation to psychological pressure.^2^

As the coronavirus pandemic rapidly sweeps across the world, it is inducing a considerable degree of fear, worry, and concern in the population at large and among certain groups in particular, such as HCWs.^1^ It is normal to feel sad, stressed, confused, scared, or angry during this crisis.^1^ HCWs experience considerable psychological distress as a result of the COVID-19 pandemic due to providing direct patient care, vicarious trauma, quarantine or self-isolation and lockdown experience.^2^

Hence, our study aims to determine the socio-psychological impact of COVID-19 pandemic among HCWs in the Nepalese Army Institute of Health Sciences.

## METHODS

A descriptive cross-sectional study was conducted from May 2020 to July 2020 at Shree Birendra Hospital, College of Medicine, College of Nursing, and College of Medical Polytechnic powered by Nepalese Army Institute of Health Sciences.

The sample size for the study was estimated based on the available email address of the HCWs. Total of 472 email address was obtained, and the sample size was determined accordingly. A set of the pre-tested self-administered structured questionnaire including the Depression, anxiety, stress and scale-21 (DASS-21) scale was sent through Google form, and the HCWs answered the questionnaire by clicking the relevant link given. A total number of 212 HCWs (both sex) have responded within the given time.

Calculating sample size,

By formula,


$$\begin{array}{ll} \text{n} & ={\text{Z}}^{\text{2}}\times \text{p}\times \text{q}/{\text{e}}^{\text{2}}\\ & =1.96^{2}\times 0.5\times 0.5/0.05^{\text{2}}\\ & =384.6 \end{array}$$

where,

Z = 1.96 at 95% Confidence Interval
p = maximum sample size estimated by taking, 50% i.e. (0.5)
q = 1 - p = 0.5
e = margin of error i.e. 5% = 0.05
N = 472

Cochrane formula for finite sample,

n'= n/[1+{(n-1)/N}]

Here n is Cochran's sample size recommendation, N is the population size, and n' is the new adjusted sample size.


$$\begin{array}{ll} = & 384.6/[1+(384.6-1)/472)]\\ = & 212 \end{array}$$

The different variables studied and analyzed were; socio-demographic information, factors related to COVID-19, impact in daily activities, preventive measures used, and spending time at home or office.

Ethical approval was taken from the Institutional Review Committee, Nepalese Army Institute of Health Sciences, (Code no: IRC/297). Likewise, formal permission from each institution's functioning under the NAIHS took informed consent from respondents. The data obtained were entered and analyzed via the Statistical Package for the Social Sciences (SPSS, version 22). Variables were defined by the frequency in numbers and percentages.

We used the DASS-21 scales to assess the subject of mental health.^3,4^ It is a set of three self-report scales deliberate in assessing the emotional circumstances of depression, anxiety, and stress. Each of the three scales comprises seven items, alienated into subscales with similar content. Items 3,5,10,13,16,17 and 21 represent the depression subscale, items 2,4,7,9,15,19 and 20 consists of the anxiety subscale, and items 1,6,8,11,12,14 and 18 represents the stress subscale. All subscales were graded on a four-point Liker scale ranging from 0 (never) to 3 (almost always). DASS-21 outcome scores are classified into four ranges: mild, moderate, severe, and extremely severe.

## RESULTS

Out of 212 HCWs, 100 (47.2%) were 20 - 30 years with mean age 34.06±10.09 years. The sex of the respondents was unevenly distributed. Female respondents were higher than male, i.e. 120 (56.6%). More than half of the respondents, 124 (58.5%) were married. Most of the respondents 186 (87.75%) were Hindu by religion, and most of the respondents, 164 (77.35%) belong to a nuclear family. Likewise, 134 (63.2%) were medical workers by profession (Table 1). Medical workers have more participated in this study because they are directly related to the treatment and prevention of COVID-19.

**Table 1:** Socio-demographic characteristics of respondents (n = 212).

Characteristics	Categories	Frequency n (%)
	21-30	100 (47.2)
	31-40	66 (31.1)
Age	41-50	30 (14.2)
	51-60	10 (4.7)
	61 and above	6 (2.8)
Sex	Male	92 (43.4)
	Female	120 (56.6)
Marital status	Married	124 (58.5)
	Unmarried	88 (41.5)
	Buddhist	22 (10.4)
Religion	Hindu	186 (87.75)
	Christian	4 (1.85)
Family type	Nuclear family	164 (77.35)
	Joint family	48 (22.65)
	Medical	134 (63.2)
Professional	Non-medical	30 (14.2)
designation	Paramedical	48 (22.6)

This COVID-19 has affected many activities of the health care worker's daily life. Towards this, 36 (17.9%) respondents had decreased their food intake, 116 (54.7%) respondents had decreased entertainment, and 64 (30.1%) had decreased work efficiency at home/office as they were in lockdown (Table 2). The lockdown caused by this COVID-19 has established a good relationship with the members of the household. About half of the respondents, 102 (48.1%) had increased and strengthened their familial relationships.

**Table 2:** Experiences of the Health Worker-related to Covid-19 (n = 212).

Impact on Daily Activities	Decreased n (%)	Increased n (%)	Did not affect any n (%)
Food Intake	36 (11.9)	98 (46.3)	18 (35.8)
Entertainment	116 (54.1)	48 (22.1)	48 (22.6)
Work Efficiency at Home/Office	64 (30.1)	90 (42.5)	58 (21.4)
Family relation at Home	32 (15.1)	102 (48.1)	18 (36.8)

The finding showed that a large number of participants are at a normal stage for depression rating scales i.e. 162 (76.4%). The percentage of extremely severe depressed participants was found to be 16 (7.5%) whereas 10 (4.7%) of them were found to have severe depression.

It has shown that 144 (67.9%) of participants were found to be normal for Anxiety status. Findings showed that the percentage for extremely severe anxiety status of the participants is more in comparison to depression. It is seen that 24 (11.3%) of the participant were extremely severe for anxiety.

Likewise, 156 (73.6%) of participants were found to be normal for Stress status. Findings showed that the percentage for extremely severe stress status of the participants is very low compared to that of depression and anxiety. It is seen that only 4 (1.9%) of the participant was extremely severe for stress (Table 3).

**Table 3:** Status of Depression, Anxiety and Stress among participants.

Depression categories	Frequency n (%)
Normal	162 (16.4)
Mild	12 (5.1)
Moderate	12 (5.1)
Severe	10 (4.1)
Extremely severe	16 (1.5)
Anxiety categories	Frequency n (%)
Normal	144 (61.9)
Mild	26 (12.3)
Moderate	12 (5.1)
Severe	6 (2.8)
Extremely severe	24 (11.3)
Stress categories	Frequency n (%)
Normal	156 (13.6)
Mild	18 (8.5)
Moderate	14 (6.6)
Severe	20 (9.4)
Extremely severe	4 (1.9)

Health care workers have also taken a variety of preventive measures in their daily life. Most of the HCWs, 208 (98%), used a mouth mask, proper hand washes, and physical distance. The HCWs also adopt other preventive measures such as; consumption of nutritious food, exercise, and enough sleeping.

We also found that more than half 111 (52.35%) respondents were talking on the phone. Because of the lockdown, they were engaged in using the phone. Very few 14 (6.6%) respondents were involved in creative writings. Besides, they used to watching television, reading books, and communicating with friends and relatives. One remarkable thing in this regard was that most of the respondents, 91 (42.92%) had cooked different food items at home. In this way, the respondents spent time at home.

Similarly, almost half of the respondents, 107 (50.47%) reported that they worked at their workplaces with regular work. But, 77 (36.32%) of respondents reported that they were directly or indirectly involved in the work related to COVID-19 (Table 4).

**Table 4:** Preventive measures adopted by Health Care Workers.

Preventive measures for COVID-19	Frequency n (%)
Use of Mouth mask	208 (98.00)
Washing hand properly	208 (98.00)
Maintaining physical distancing	208 (85.48)
Consumption of nutritious food	16l (18.11)
Enough sleeping	154 (12.64)
Physical exercise	130 (61.32)
Spend time during lockdown at home	
Hanging on mobile	111 (52.35)
Physical exercises	101 (50.41)
Creative writings	14 (6.6)
Watching TV	10 (33.01)
Cooking at home	91 (42.92)
Reading books	102 (48.11)
Communication	91 (45.15)
Spend time during lockdown at office	
Work regularly as before	101 (50.41)
Working related to COVID 19 issues	11 (36.32)
Regular work and	19 (31.26)
Unrelated work	4 (1.88)

## DISCUSSION

The first studies on the impact of the coronavirus pandemic on health professionals were developed in China but, as the pandemic spread, other countries started to publish cross-sectional studies analyzing the psychological response of healthcare workers to the pandemic.^5-7^ Most studies reported a high prevalence of anxiety (ranging from 30% to 70%) and depressive symptoms (20-40%)^8-10^ Insomnia, burnout, emotional exhaustion or somatic symptoms were also similarly reported.^8,11,12^

In this background, this study was conducted to evaluate the prevalence of depression, anxiety, and stress among the HCWs on duty. For this study, HCWs included doctors, health assistants, laboratory assistants, paramedics, administrative staff, staff nurses, sanitization workers, ward attendants, security guards, and ambulance drivers who are directly or indirectly involved in the care of patients with COVID-19. Our survey found that there are many factors that directly or indirectly related to COVID-19. As this study was conducted on health care workers, it is important to mention here what factors have influenced their mobility and the nature of their work or how they deal with the situation during this lockdown period.

Several socio-demographic variables like age, gender, religion, marital status, profession, place of work, department of work and psychological variables like poor social support, self-efficacy, travel history, contact history, families or themselves were associated with increased stress, anxiety, depressive symptoms, insomnia in HCWs were analyzed. This study suggests that 12.2% of the HCWs on COVID-19 duty in the Nepalese Army Institute of Health Sciences are suffering from severe depression, followed by 14.1% severe anxiety and 11.3% severe stress. A study from China suggests that about half (50.4%) of the HCWs are reported symptoms of depression, 44.6% had anxiety symptoms, 34% had insomnia, and 71.5% reported distress.^13^

Another recent meta-analysis of studies reported the pooled prevalence of anxiety to be 23.2% and that for depression to be 22.8% and the findings of the present study are within this reported range.^14^ This high level of depression, anxiety, and stress among the HCWs in NAIHS could be attributed to factors like overwhelming health care system, prolonged work shift, lack of Personal Protective Equipment (PPEs) and added fear of infection to self or family. Previous studies on outbreaks and epidemics of various infectious diseases, i.e. SARS-CoV-1, H1N1 influenza, and Ebola virus proved to cause significant short and long term psychological impact on HCWs in the frontline. These studies have also linked mental morbidity in HCWs to the inadequacy of PPEs and increased the risk of infection exposure.^15^

Our study observed that the respondents' contact history with the COVID patient is the most influencing factor that affected their mental status. Especially in this, HCWs have direct contact with COVID patients psychologically more affected than indirect contact in all manners of psychology, i.e. depression, anxiety, and stress. Likewise, female respondents appeared to be more prone to depression, anxiety, and stress than men respondents, which may be related to their sensitivity to psychology. Some factors such as; fear of being infected, the inadequacy of PPE, increased risk of exposure to infection, lack of proper training, and increasing numbers of hospital cases for COVID-19 may be the main reasons for contact history for the psychological impact of health care workers.

Therefore, during the pandemic, the majority of HCWs have experienced unpleasant emotions, including fear, hyperarousal, intrusive memories, and insomnia, as well as some related to sadness or emotional exhaustion. The more they were exposed to unexpected life-threatening situations or uncertainty, the more mental distress they were likely to experience. However, most HCWs have chosen to take care of patients with COVID-19 infections despite the risk to themselves and their families.

The study is bounded with time so that it could not assess the impact of Covid-19 in our study population. This study only assessed the current status of Depression, anxiety, and Stress among the respondents. Also, it could only include the participants from NAIHS administration.

## CONCLUSIONS

The current COVID-19 pandemic has affected the psychological wellbeing of health care workers. This has imposed a significant level of depression, anxiety, and stress on health care workers caring for infected patients, with their main concern being the risk of transmitting the infection to their families or acquiring it themselves. To conclude, this study highlighted that the HCWs who are an integral part of the front line warriors to fight against the pandemic are suffering from psychiatric morbidity.
